# Applications of Next-Generation Sequencing Technologies and Statistical Tools in Identifying Pathways and Biomarkers for Heat Tolerance in Livestock

**DOI:** 10.3390/vetsci11120616

**Published:** 2024-12-02

**Authors:** Gajendirane Kalaignazhal, Veerasamy Sejian, Silpa Mullakkalparambil Velayudhan, Chinmoy Mishra, Ebenezer Binuni Rebez, Surinder Singh Chauhan, Kristy DiGiacomo, Nicola Lacetera, Frank Rowland Dunshea

**Affiliations:** 1 Rajiv Gandhi Institute of Veterinary Education and Research, Kurumbapet 605009, Puducherry, India; gnazhal99@gmail.com (G.K.); mv.silpa@gmail.com (S.M.V.); binunirebez.e@gmail.com (E.B.R.); 2 Department of Animal Breeding and Genetics, College of Veterinary Science and Animal Husbandry, Odisha University of Agriculture and Technology, Bhubaneshwar 751003, Odisha, India; cmishra@ouat.ac.in; 3 School of Agriculture, Food and Ecosystem Sciences, Faculty of Science, The University of Melbourne, Melbourne, VIC 3010, Australia; ss.chauhan@unimelb.edu.au (S.S.C.); kristyd@unimelb.edu.au (K.D.); 4 Department of Agriculture and Forest Sciences, University of Tuscia, 01100 Viterbo, Italy; nicgio@unitus.it; 5 Faculty of Biological Sciences, The University of Leeds, Leeds LS2 9JT, UK

**Keywords:** biotechnological tools, biomarkers, heat stress, next-generation sequencing, thermo-tolerance

## Abstract

The climate change-associated abnormal weather patterns negatively influences the productivity and performance of farm animals. Heat stress is the major detrimental factor hampering production, causing substantial economic loss to the livestock industry. Therefore, it is important to identify heat-tolerant breeds that can survive and produce optimally in any given environment. To achieve this goal, a clearer understanding of the genetic differences and the underlying molecular mechanisms associated with climate change impacts and heat tolerance are a prerequisite. Adopting next-generation biotechnological and statistical tools like whole transcriptome analysis, whole metagenome sequencing, bisulphite sequencing, genome-wide association studies (GWAS), and selection signatures provides an opportunity to achieve this goal. Through these techniques, it is possible to identify permanent genetic markers for heat tolerance, and by incorporating those markers in marker-assisted breeding selection, it is possible to achieve the target of breeding for heat tolerance in livestock. This review gives an overview of the recent advancements in assessing heat tolerance in livestock using such ‘omics’ approaches and statistical models. The salient findings from this research highlighted several candidate biomarkers that have the potential to be incorporated into future heat-tolerance studies. Such approaches could revolutionise livestock production in the changing climate scenario and support the food demands of the growing human population.

## Simple Summary

Climate change-associated heat stress negatively impacts farm animals’ productive and reproductive performances, leading to huge economic loss. To identify/develop ideal heat-tolerant breeds, understanding genetic differences and molecular mechanisms is crucial. Next-generation biotechnological and statistical tools like transcriptome analysis, whole metagenome sequencing, bisulphite sequencing, genome-wide association studies (GWAS), and selection signatures can help identify permanent genetic markers for heat tolerance. These markers can be incorporated into marker-assisted breeding selection to achieve heat tolerance in livestock. Recent advancements in assessing heat tolerance in livestock using omics approaches and statistical models highlight potential biomarkers for future studies, potentially revolutionising livestock production and supporting the growing human population.

## 1. Introduction

It has been estimated that, with the increasing atmospheric greenhouse gas concentration scenario, global temperature will drastically increase between 2.6 and 4.8 °C by 2081–2100 [1], causing severe global warming. This projected global warming effect may cause alarming impacts on animal species due to abnormal weather patterns [2].

Climate change contributes to multiple environmental factors that could negatively influence farm animals’ productive and reproductive performance [3]. Among the various climate change-associated factors, heat stress is established to have a detrimental role in hampering livestock production especially in developed countries as the predominantly rear exotic breeds in comparison to the adapted breeds in developing countries. This could cause severe economic loss to the livestock sector in developed countries. However, the production loss could be incurred even in adapted breeds but to a less impactful magnitude as compared to the exotic breeds. These findings are very substantial from the perspective of developing countries, given that livestock contributes enormously to these countries’ economies. The economic loss incurred due to climate change could be attributed to reduced growth, milk, meat, reproductive performances, and disease occurrences [4]. However, species and breed variation were found for the above-mentioned adverse effects of climate change [5]. Upon comparative analysis of heat tolerance, it has been observed that small ruminants are more tolerant. High-producing animals, especially dairy cattle, were found to be more sensitive to the effects of heat stress [6] compared to other livestock species. The fact that dairy cattle are more susceptible to heat stress is because of their high metabolic rate, due to high milk production that in turn results in increased internal heat generation. Additionally, their vulnerability to environmental stresses is further increased by the physiological demands of lactation, and heat stress frequently results in decreased feed intake, which affects their nutritional requirements [7]. This signifies the importance of establishing more thermo-tolerant breeds to sustain livestock production in the changing climatic scenario by understanding the genetic differences and molecular mechanisms.

The recent tremendous progress achieved in advanced molecular tools comprising next-generation sequencing (NGS) technology offers scope to meet all these critical goals for the livestock sector [8], offering a perpetual solution. The NGS is a DNA-sequencing technology performed by sequencing multiple small fragments of DNA in parallel, either by sequencing the entire genome or specific areas of interest [9]. The NGS is an advanced molecular technique that plays a vital role in recognising heat-tolerant animals, which helps transform the animal breeding industry by improving their productivity and reproductive efficiency [10]. By redefining the breeding policy, it is possible to breed for specific traits governing different productive and adaptive functions. Through NGS, it is possible to identify various pathways which are significantly altered during heat stress exposure in livestock. By validating important traits in these significantly altered pathways, it is possible to identify potential biomarkers governing productive and reproductive functions. These markers could be used to redefine the breeding policy using marker-assisted selection to develop new thermo-tolerant breeds with improved productive and reproductive capacity. The modern era has led to the development of various biotechnological tools and statistical models, which include the microarray technology, whole transcriptome analysis, whole metagenome sequencing, bisulfite sequencing, genome-wide association studies (GWAS), and selection signature.

The combination of these biotechnological tools and traditional or conventional breeding selection paved the way for the emergence of marker-assisted breeding Selection (MAS), a specialised method for identifying superior/better-performing animals [11]. The MAS is an indirect selection approach where marker genes are employed to identify the presence of desirable genes. They have a wide range of applications and can be used to identify thermo-tolerant genes associated with production, reproduction, immune response and adaptation, thereby improving the animal’s growth, milk and meat quality, immune response, and thermo-tolerant capacity. These identified biomarkers could be incorporated into the existing breeding programmes through MAS. Such an approach may help to develop more climate-resilient livestock breeds as well as identify heat-tolerant individuals in the existing breeds. These identified breeds may have the potential to produce optimally in addition to having the potential to survive adverse environmental conditions. However, most research demonstrates that heat tolerance is polygenic, and MAS is not the best tool to improve it. This literature review is an effort to collate and synthesise information about climate change-associated economic loss in the livestock sector and the necessity for identifying potential pathways and biomarkers associated with heat tolerance in livestock through the applications of NGS technologies and statistical tools as the best possible solution for sustainable livestock production.

## 2. Climate Change and Heat Stress Significance

According to the IPCC (Intergovernmental Panel on Climate Change) 2018 report, the world is set to reach the 1.5 °C temperature increase level between 2030 and 2052, resulting in climate change. It is predicted that only drastic cuts in carbon emissions will help prevent environmental disasters [12].

The IPCC defines climate change as a change in the state of the climate that can be identified by changes in the mean and/or the variability of its properties that persist for an extended period, typically decades or longer. It can be either due to natural, internal processes or external forces causing various detrimental effects like melting of the glaciers from the poles, changing rainfall patterns, ocean acidification, drought, etc., affecting all global economic sectors. Climate change emerges as a major threat to the ecosystem, which is expected to cause frequent heat waves, especially in tropical regions, causing distress to livestock and human populations [13]. In many parts of the world, especially in developing countries, climate change is viewed as a serious threat to ecosystems, livestock production systems, and the survival of several species. The demand for animal products worldwide is anticipated to double in the first half of this century due to increasing affluence and human population expansion [3]. The effects of climate change can be direct or indirect, where the direct effects are mainly due to environmental factors that affect livestock health. In contrast, the indirect effects are linked to alterations in the distribution and survival of vectors/pathogens [14].

Among the stressors occurring due to climate change, heat stress is found to be the most harmful, having a negative impact on animals’ growth rate, immune response, milk production, meat production, reproductive performance, and disease occurrence as a result of drastic biological changes in the body due to stress causing enormous economic loss [15]. Though animals’ responses to heat stress vary among species, age, and sex, an individual’s genotypic trait is of more significance as the response to heat stress differs between them [16]. Thus, it is evident that both direct and indirect effects of climate change ultimately affect a country’s economy. This paves the way for challenges in all parts of the globe, especially in livestock production, as it forms an important economic and ecological role in the agricultural systems and livelihood of marginal farmers.

## 3. Economic Implications of Heat Stress in the Livestock Sector

Sustainability in the livestock sector is a topic of concern, particularly in tropical regions where many developing countries are located [17]. This is due to the strong connection between agro-climatic conditions, population density, cropping systems, and livestock production [18]. The high humidity and temperature in tropical regions lead to heat stress in animals, causing an imbalance between heat production and dissipation [19]. Heat-stressed animals experience reduced productivity, primarily due to increased maintenance of body temperature and altered feed intake [20]. Heat stress also negatively impacts reproductive systems in both males and females, affecting cellular functions and impairing the reproductive organs [17].

Among all the species, high-yielding dairy cattle are the most sensitive to the changing climatic conditions, which alter both the quality (organic composition and inorganic composition) and quantity of milk and milk products [21]. Berry et al. [22] formulated an equation to calculate the decline in milk production, which clearly stated that there will be a decrease in daily milk yield when the animals are moved out of the thermo-neutral zone because of the increased temperature–humidity index [23]. The stage of lactation also plays an important role in deciding the amount of milk reduction where cows in mid-lactation were found to be more sensitive with a 35% reduction [17], causing huge economic loss. However, heat stress in beef cattle is usually less severe when compared to dairy cattle. In 2003, St. Pierre reported that heat stress had a negative economic impact on dairy and beef industries resulting in a loss of $897 million and $369 million per year, respectively [24]. Regarding meat quality, acute heat stress results in pale, soft, and exudate (PSE) meat, while chronic stress causes dark, firm, and dry (DFD) meat. In addition, meat quality due to heat stress is hampered because of oxidative stress and lipid and protein oxidation, leading to reduced shelf life of meat [25]. However, the meat quality of other species is also affected, and the impact of heat stress varies across species.

Heat stress impairs the animal’s immune system, making it susceptible to infectious diseases. Heat stress weakens the animal because of the shift in immune function from cell-mediated to humoral immunity [26]. The immune responses depend upon the duration of exposure, where chronic stress leads to the suppression of innate and adaptive immune response, whereas acute stress may be either an immune-modulator or immunosuppressive [27]. In 1985, Giesecke reported that udder infections and the incidence of mastitis increase during the summer because of a decline in defence mechanisms due to heat stress [28].

Thus, exposure to climatic changes, especially heat stress, negatively impacts the animal’s health, productivity, and product quality. This causes substantial economic losses to the livestock industry due to slow growth rates, reduced fertility, increased veterinary costs, inconsistent carcass quality and composition, reduced market weights, and increased animal welfare issues [25]. St. Pierre reported that heat stress results in an estimated annual economic loss to livestock industries of between $1.69 and $2.36 billion. Of these, $897–$1500 million occurred in the dairy industry, $370 million in the beef industry, $299–$316 million in the swine industry, and $128–$165 million in the poultry industry [24]. Of course, these estimates are now two decades old and so economic losses are expected to be much greater. Sometimes, heat stress can even lead to the animal’s death; therefore, it is essential to consider heat stress as an important factor compromising animal health. All this necessitates the urgent need to find a solution to tackle the economic losses.

## 4. Importance of Identifying Heat-Tolerant Animals

Despite the trend towards intensification, the increase in demand for animal products will likely continue to be a major factor over the coming decades, resulting in a net expansion of the area used for livestock. Due to the increase in human populations, it is predicted that by the year 2050, the world’s food demand will double. The changing climate scenario poses a serious threat to food security by challenging the natural adaptive capacity of livestock, thereby affecting the animal’s productivity [29].

Livestock plays a crucial role in the economy, especially in tropical, developing countries. It contributes to about 80% of the gross domestic product (GDP) in dry lands and supports the livelihoods of a billion people [30]. Additionally, livestock contributes 40% to the worldwide value of agricultural output, ensuring food security [31]. In India, the livestock sector contributes substantially to the country’s GDP, accounting for 4.1% of the total GDP [32]. Increasing the productivity of the tropical livestock sector is essential to meet the growing demand for animal-sourced foods and to reduce poverty. Smallholder livestock keepers manage a substantial portion of agricultural land in the tropics and offer the potential for improving livelihoods and environmental stewardship [33].

Livestock production is affected mainly by three factors: climate, nutrition, and health, of which climate plays a substantial role [34]. It is crucial to develop heat-tolerant breeds to adapt to changing climatic conditions via proper identification of thermo-tolerant animals, and those animals can be selected and used for further breeding programmes. Studies have shown that indigenous livestock breeds in developing countries are more tolerant to extreme climates due to their physiological and genetic adaptations than exotic European breeds [35]. These indigenous breeds survive and perform better when exposed to extreme heat stress, water scarcity, and reduced pasture availability, maintaining their reproductive potential [3]. Due to the genotype–environment interaction, the zebu cattle of India are highly adapted to the heat stress condition by reducing their metabolic rate, heart rate, and high sweating capacity, but their production is considerably low when compared to exotic breeds as high production requires high metabolic activity and energy expenditure [36].

However, differences exist in the adaptability between the indigenous breeds as there are genetic variations among them [37]. In 2018, Pragna reported that there exists variation in the growth performance between indigenous goat breeds when exposed to summer heat stress and proved that the Salem black breed performs much better compared to the Osmanabadi and Malabari breeds, indicating the breed’s enhanced capacity to deal with the challenges posed by heat stress [38]. Thus, if the genes responsible for thermo-tolerance and disease resistance were identified, it would be possible to transfer them from the indigenous breed into the improved breed, thereby producing livestock that will have an increased production potential and is resistant to endemic diseases. Therefore, it is essential to find the most appropriate thermo-tolerant model, which can be a way forward for sustainable livestock production. Breeding techniques must guarantee the selection of livestock breeds suitable for a given agro-climatic zone with superior thermos-tolerance, drought tolerance, and the capacity to thrive in sparse pastures. As a result, it is essential to evaluate the relevant species in the climates that they will probably be exposed to. Therefore, animal scientists have an impending challenge in properly identifying thermo-tolerant breeds and enhancing the resilience of livestock to climatic variability as it will help lift the livelihood of marginal farmers in the tropics as most of the population there depend upon livestock for their daily livelihood.

## 5. Difference in Thermo-Tolerance in Major Ruminant Species

The susceptibility of livestock to heat stress varies according to species, genetic potential, life stage, management or production system, and nutritional status [17]. Building a livestock’s adaptive capacity is essential for boosting the thermo-tolerance of livestock species; therefore, it is important to develop an ambitious strategy to address the key factors that influence this capacity [39].

### 5.1. Physiological and Behavioural Responses

The adaptive capacity of livestock is determined by physical and genetic traits that allow them to survive in challenging environments. Animals can adapt through changes in appearance, behaviour, and genetic makeup, which occur gradually over generations. Out of various adaptation responses, an animals’ primary method of reducing heat load is behavioural adaptation [40]. The major behavioural responses include feeding, defecating and urinating frequency, water intake, lying time, standing time, shade-seeking behaviour, and increased frequency of drinking [41]. Other responses include open-mouthed panting, increased salivation [42], and nighttime lying rumination pattern [41]. Among these, shade seeking is one of the most immediate and substantial behavioural changes observed in animals under heat stress. Dairy cattle seek shade in warm conditions, and this behaviour is found to occur more frequently as air temperature and solar radiation rise [43], but buffaloes seek shade only in the absence of wallows. Such differences could be due to the differences in their physiological and anatomical adaptability, wherein buffaloes have poor cutaneous heat dissipation [44]. Reduced feed intake is another considerable and well-documented behavioural response to increased heat load in ruminants. Studies have shown that agricultural animals such as cattle, sheep, and goats consume less feed throughout the summer [40,45]. Shilja et al. [40] and Aleena et al. [46] confirmed that increased water usage in heat dissipation through skin and respiratory water evaporation is closely related and results in high water consumption. Substantiating these studies, Markwick [47] reported that sheep consume more water for evaporative cooling under hot weather conditions. The defecation and urination frequency alters with changing environments, which substantially decreases in goats [41] and buffaloes [48] who are subjected to heat stress. The other behavioural response exhibited in heat-stressed animals is increased standing time, and a study reports an increase of 10% in dairy cattle when the heat load increased by 15%, as an attempt to avoid being exposed to the ground’s increased heat load and to facilitate easy heat drainage from the body [49]. By standing longer to escape the conductive and radiative heat from the hot ground surface, the animals increase the evaporative heat loss from the body surface and aid in convection. Buffaloes withstand the heat stress by wallowing. The wallowing behaviour lengthens the buffaloes’ grazing period because it eases their discomfort from the heat. This thermoregulatory strategy shields them from solar radiation and offers a cooling effect [41].

Ruminants’ major physiological response to high heat load is increased respiratory and perspiration rates [50,51]. Usually, as the ambient temperature rises, animals’ respiration rates (RRs) and sweating rates increase. By vaporising more moisture into the environment, the respiratory and cutaneous cooling systems directly include the dissipation of the additional heat load in the body [21,52,53]. When subjected to high heat stress, various cattle breeds [54], goats [13], and buffaloes [48] showed an increase in RRs. According to Leite et al. [55], Morada Nova ewes that have become accustomed to the Brazilian weather are more tolerant to hot, dry circumstances, and can sustain normal respiratory rates at an ambient temperature of 32 °C. Sweating and panting are the thermoregulatory mechanisms that greatly influence the heat exchange between the animal and its surroundings. Weather factors that affect sweating include wind speed, air temperature, relative humidity, and sun and heat radiation [39]. In an experiment with dairy cows, Hillman et al. [56] found that black cows sweated more (800 W/m^2^) than white cows (500 W/m^2^). Furthermore, Da Silva et al. [57] demonstrated that light-haired coats had better reflectivity than dark-haired coats for wavelengths between 300 and 850 nm. A study conducted by Wanker et al. [48] on thermoregulatory and adaptive responses of adult buffaloes during hyperthermia revealed that panting and sweating were higher during heat stress.

Apart from these adaptive responses, morphological characteristics in cattle are crucial because they have a direct impact on the heat exchange mechanisms (cutaneous convection, radiation, and evaporation) between the animal and its environment [58]. One of the key morphological characteristics that gives heat-stressed animals the ability to adapt is coat colour. Additionally, coat thickness, length, and hair density may influence adaptation. Cutaneous evaporation is recognised as a substantial method for heat dissipation in cattle [3]. Indigenous sheep that have successfully adapted to arid and semi-arid climates include morphological characteristics like carpet-type wool, which better shields irradiation from the sun and permits efficient cutaneous evaporation and dispersion of heat [59]. In addition, animal size, shape, and surface area are considerable morphological adaptations affecting an animal’s ability to regulate body temperature [51]. The noticeable fat tail is also acknowledged as a morphological adaption for sheep in improving heat transfer [60]. Buffaloes have less tolerance to heat stress than cattle due to the presence of a black coat colour and fewer sweat glands [48].

Another key strategy animals use to combat heat stress is metabolic adaptation, which essentially involves lowering metabolic heat output [38]. Thyroid hormones like triiodothyronine (T3) and thyroxine (T4) are recognised as indicators for evaluating the thermo-tolerance of farm animals and play a considerable role in controlling thermogenesis [61] and maintaining metabolic adaptability [62]. When subjected to heat stress, the concentrations of T3 and T4 are likely to drop because of the direct impact of heat stress on the hypothalamic–pituitary–thyroid axis to decrease the generation of thyrotropin-releasing hormone. A recent study also confirmed the decrease in serum and plasma concentration of T3 and T4 in heat-stressed goat, sheep [3], heifers, and buffaloes [48].

### 5.2. Cellular and Molecular Responses

In livestock, heat stress causes various cellular and molecular reactions [63]. For instance, specific genes’ expression patterns are altered at the cellular level in animals, which are essential for thermos-tolerance [64]. Upon exposure to heat stress, it is thought that the classical heat shock protein (*HSP*) genes, apoptotic genes, various cytokines, and toll-like receptors are upregulated. In addition, numerous other genes like superoxide dismutase (*SOD*), nitric oxide synthase (*NOS*), thyroid hormone receptor (*THR*), and prolactin receptor (*PRLR*) [65] are also linked with thermos-tolerance. A family of heat shock transcription factors (HSF) controlled by the inducible expression of *HSF* genes influence the cellular response to thermal stress in mammalian animals at the transcriptional level.

Among various HSPs, the development of thermo-tolerance in livestock species mostly correlates with HSP70 and HSP90. According to reports, HSP70-1 and HSP70-2 are the most prevalent and temperature-sensitive forms. Sheep, buffalos, cattle, broilers, and goats were shown to have elevated levels of HSP70 and HSP90. HSP70 has been proven to have cytoprotective properties in various organs, including the gut, kidney, and cattle embryo. The most prevalent member of the HSP family, *HSP70*, is essential for goats’ ability to adjust to environmental stress and heat. During hyperthermic stress, goats exhibit increased expression of numerous HSPs, including *HSP32*, *HSP40*, *HSP60*, *HSP70*, *HSP90*, *HSP110*, and many others [66]. Due to their high capacity to produce, survive, and breed in the context of climate change, goats are considered to be the most adapted species among other livestock species [67].

## 6. Biotechnological Tools to Identify Climate Resilience in Animals

With the advancement in genomic analysis, high-throughput methods, now referred to as NGS technologies, perform massive parallel sequencing of millions of DNA fragments from a single sample [9]. In recent years, partial or complete sequencing of the genomes of various domesticated livestock species, including chickens, pigs, cattle, sheep, goats, and horses, has been achieved [68]. The detection of molecular markers in DNA fragments found in the genome is the primary focus of these modern molecular technologies [69], thereby improving the accuracy of the selection and accelerating genetic gain in the population [70]. Genes and chromosomal areas that are more susceptible to the effects of the environment could be identified by utilising NGS technologies which reveals the considerable gene–gene and gene–environment interactions that boosts the rate of genetic improvement [68]. The NGS technologies are the most realistic approaches for the future of livestock production and paves the way to boost animal’s tolerance to heat stress, lower methane emissions from cattle and sheep, improve disease resistance, lessen environmental effects, reduce the competition for land and water, and finally, boosts the productivity of animals [71].

From the previous discussions, thermo-tolerant animals have to be produced to sustain the livestock sector, which is a key asset of nations globally. Therefore, identifying the potential genetic markers linked to thermo-tolerance would play a vital role in understanding the molecular and cellular mechanisms of the animal’s thermal tolerance in response to heat stress [72]. It is now possible to identify thermo-tolerant animals by assessing their molecular responses using biotechnological tools and statistical models like whole genome resequencing, transcriptome analysis, whole metagenome analysis, bisulphite sequencing technology, GWAS, and selection signatures, which will be dealt with in detail in the following sections.

### 6.1. Whole Genome Resequencing

Whole genome resquencing (WGS) is a potential biotechnological tool that enables thorough analysis of genetic variations like single nucleotide polymorphisms (SNP), insertions and deletions (INDELs), copy number variations (CNV) and structural variations (SV) [73,74,75,76]. WGS can give in-depth information covering all minute variations that are genetically driven which may be difficult to study using commercially available chips. Thus, WGS is an advanced methodology to quantify even minute differences in genetic variations which could help to identify useful traits which may not be possible using conventional methodologies. Such detailed information can help in identifying new traits that can be used as potential markers for strategizing breeding programmes. This approach maybe highly beneficial for identifying potential thermotolerant breeds with optimum productive efficiency. The WGS makes it possible to accurately identify and characterise these genetic variations, shedding light on genetic diversity, evolutionary links, and the underlying mechanisms of complex traits in livestock. Numerous studies have been reported that helped in understanding these variations linked to heat stress adaptation, thereby helping to improve the resilient potential in animals to adapt to harsh climatic conditions [75,76,77,78,79].

#### 6.1.1. Single Nucleotide Polymorphism

Single Nucleotide Polymorphisms (SNPs) are bi-allelic genetic markers. They are abundant throughout the genome and are simple to assess and interpret. The SNPs could capture the linkage disequilibrium (LD) information in the genome with appropriate coverage and density over the entire genome to identify the genes responsible for a particular trait. In the livestock sector, SNPs help us better understand domestication, breed creation, and species evolution, creating novel population genetics hypotheses, analysing the genetic underpinnings behind sophisticated agricultural features, and enhancing methods of selection for improved animal productivity [80].

The SNPs offer tools for performing association studies and identifying genetic markers for the selection of considerable traits [81]. They have been extensively used to identify genetic markers related to production traits [81], reproduction traits [82,83], adaptative traits [84,85,86,87,88], and immune response [89] associated with thermo-tolerance in animals.

The SNPs associated with reproductive traits in livestock have also been explored. Huang et al. [82] detected five SNP sites in the 5′-flanking regions of the *HSP 70.2* gene. They reported its close association with the semen quality of pigs (Duroc, landrace and Yorkshire) during the hot season. Likewise, Said and Putra [83], in their experiment on Pasundan cattle, detected eighteen SNP sites in the 5′UTR region of HSP 70, and among them, two SNPs (g.1117G/A and g.1125A/C) were concluded as potential biomarkers as these animals had better services per conception rate during heat stress when compared to other animals. Thus, these SNPs can be used as genetic markers for breeding to improve heat-tolerance and reproductive performances in animals. Abbas et al. [81] detected polymorphisms in the *HSP70* gene, which validates its association with metabolites like lactate (C181T) and lipid peroxide (SNP A72G) during heat stress, while genes like dopamine (SNP A12G) and superoxide dismutase (SNP C131G) were expressed under cold stress in Holstein cattle.

A recent study using Chinese cattle revealed a significant SNP on the gene *MYO1A* that had close association with thermo-tolerant traits. The authors concluded it to be a potential biomarker for improving the resilience capacity in animals as the SNP showed a close association with the climatic variables [86]. Similarly, Bai et al. [87] performed whole-genome resequencing in tropical chicken breeds that helped them to identify several potential genes linked to adaptation traits. The authors were additionally able to identify the pathways and functions of these genes and validated them to be potential markers for improving the adaptation capacity to tropical conditions in these chicken breeds [87]. Another whole genome sequencing study on indicine cattle revealed several genomic regions linked to thermo-tolerance. This aided in a better understanding on the evolution of indigenous cattle breeds to adapt to the harsh tropical climate [88].

In 2017, Yakubu et al. [89] conducted research in three Nigerian tropical goat breeds (West African dwarf, Red Sokoto, and Sahel) that had varied genetic potential for heat tolerance at the *DRB* (D-related beta chain) gene. They studied the gene polymorphism in the second exon of the *DRB* gene. They found that the SNPs in this study had a great chance to be utilised as markers to determine the animals susceptible to or resistant to a particular tropical disease. Similarly, Liu et al. [90] reported a novel SNP in exon 17 of the *ATP1A1* gene that had a considerable association with the thermo-tolerant capacity of Holstein cattle and indicated that the cows had higher efficient transcription and a stronger ability to adapt to the heat stress environment. In another study on chicken breeds (Huainan and Wenchang), Wan et al. [91] detected an SNP in exon 14 of *HSP90B1* that had the potential to be used in breeding for improved heat tolerance in chickens.

In farm animals, *HSBP1* (heat shock factor binding protein) is a potential gene for thermo-tolerance. Wang et al. [92] revealed that Chinese Holstein cattle with the H2H2 haplotype combination of the *HSBP1* gene had improved thermo-tolerance. However, Saikia et al. [85] detected polymorphism in the *HSBP1* of Murrah buffaloes. They concluded that the SNP had no substantial association with the heat-tolerant traits (respiration rate, rectal temperature, and heat-tolerance coefficient). Thus, further research must be conducted to conclude the role of the *HSBP1* gene in thermoregulatory function in animals.

#### 6.1.2. Insertions and Deletions (INDELs)

Indels (insertion and deletions) are short genetic variations that occurs when nucleotides are added to or deleted from a DNA sequence. Gene function can be profoundly affected by INDELs, which may damage regulatory areas or coding sequences, changing the synthesis or function of proteins [93]. To understand the underlying mechanism of adaptation in indigenous cattle, Vijayakumar et al. [75] performed comparative whole genome sequencing analysis in five cattle breeds of India. They identified 155,851,012 SNPs and 10,062,805 INDELs across the whole genome that were linked to pathways associated with thermo-tolerance and adaptation mechanisms. Additionally, the authors found that the putative thermo-tolerant genes were related to feed intake, metabolism, osmotic stress response, oxidative stress response, sweating, skin and hair characteristics, and heat shock response. Similarly, Rosse et al. [77] identified 2,676,067 SNPs and 463,158 INDELs while analysing the whole genome sequence of Guzera cattle. The results of functional analysis revealed the genes belongs to pathways associated with environmental adaptation traits, immune system, sensory perception, signal transduction, environmental adaptability, and cell communication.

#### 6.1.3. Copy Number Variations (CNV)

Copy number variations is another important form of genetic variation that involves repeated genomic sequences [94]. Several studies have been reported for identifying CNVs that have aided to improve the thermo-tolerant potential in livestock. A study conducted by Salhein-Dehkordi et al. [78] in sheep population identified several CNVs that can be used as biomarkers to select for individuals with better adaption potential. They detected functional candidate genes for heat stress and cold climate adaptation such as *UBE2L3*, *B3GNTL1*, *SHANK2*, *TRAF2*, *COPG1*, and *GTF2F1* associated with the identified CNVs. Similar study was conducted in Kholmogory and Yakut cattle breeds that led to the identification of CNVs responsible for the adaptation of these animals. The authors suggested that the enrichment of several fatty-acid-related genes implicated in lipogenesis and growth traits could be the reason for their potential involvement in thermoregulation in these animals [95].

#### 6.1.4. Structural Variations (SV)

Structural variations are alterations in the DNA region that can profoundly change the structure and function. These modifications include inversions, translocations, duplications, and deletions [96]. Ben-Jemma et al. [76] using whole genome sequencing detected structural variation in Creole cattle that were responsible for the adaptation of these animals to the hot and humid climate. They identified a total of 32,821 structural variations in which 6639 SVs were identified for the first time. The newly identified structural variant regions include three duplications and three deletions. The authors stated that the genes were linked to the metabolic process of tRNA threonylcarbamoyl adenosine, which is crucial for temperature adaptation in thermophilic organisms. This suggests a potential role for the tRNA metabolic process in the thermo-tolerance of Creole cattle from Guadeloupe to tropical climate. Another study examined the SVs linked to adaption traits in cattle from Hainan and Mongolia. In these East Asian cattle breeds, the authors found SVs linked to pathways for disease resistance and climate adaptability [79].

Therefore, the above studies prove that whole genome resquencing technology can be used as a potential biotechnological tool to identify potent markers through the identification of the genetic variations that could be incorporated in breeding programmes to improve livestock’s resilience to heat stress.

### 6.2. Transcriptome Analysis

RNA is a polymeric molecule involved in many biological functions, including the coding, decoding, control, and expression of genes. The complete set of RNA transcripts in each cell for a particular developmental stage or physiological condition is known as the transcriptome [97]. Whole-transcriptome shotgun sequencing is the process of analysing a sample’s RNA content and composition using high-throughput sequencing technology [98]. Transcriptome analysis is robust, and to interpret the functional components of the genome and to comprehend the underlying mechanisms of development and disease, a thorough understanding of the transcriptome is necessary. Microarray technology has been quickly substituted with RNA sequencing (RNA-seq) because of its improved resolution and increased reproducibility [99].

Transcriptome sequence constitutes a meaningful resource for developing many popular molecular markers such as SNPs and microsatellites. If the application necessitates the use of multiple markers but full sequencing is not financially feasible, the transcriptome offers a valuable functionally relevant subset of the genome [98]. This application helps in the identification of tissue-specific gene expression changes during heat stress. Several studies have explored the potential of whole transcriptome analysis in identifying the thermo-tolerant capacity in animals.

Gao et al. [100] studied the transcriptomic profile of mammary tissue of Holstein cows and explained the mechanism for reduction in the milk protein synthesis during heat stress. They found heat stress affected more than 2777 genes in the mammary tissue and an overall decrease in the mammary tissue’s metabolic activity. They observed the downregulation of milk protein-encoding genes and several key genes related to the regulation of protein synthesis and amino acid and glucose transport along with the upregulation of genes like tumour necrosis factor (*TNF*), interferon gamma (*IFNG*), and insulin-like growth factor (*IGF-1*), which are involved in immune function and inflammation. This explains that the decline in milk protein synthesis during heat stress could be because of the general decline in metabolic activity, which could be attributed to an increased inflammatory response from an intrinsic transcriptomic alteration. Yue et al. [101] reported similar results on the downregulation of immune response genes and casein gene expression, which led to a decrease in milk production under heat stress. Another study was conducted in mammary epithelial cells (MECs) of Riverine buffaloes subjected to heat stress in vitro [102]. They observed cellular apoptosis, necrosis, and an overall decrease in MEC cell viability and proliferation. The transcriptomic profile revealed one hundred and fifty three upregulated and eight downregulated genes that could be termed heat-responsive genes in buffalo MECs.

Liu et al. [103] identified six hub genes (*IL18RAP*, *IL6R*, *CCR1*, *PPBP*, *IL1B*, and *IL1R1*) related to heat tolerance involved in cytokine–cytokine receptor interaction while studying the mRNA and miRNA expression profile data between heat-tolerant and non-heat-tolerant crossbred buffaloes (Nili Ravi × Murrah). The study revealed 753 differentially expressed genes (DEGs) and 16 differentially expressed miRNAs between them, of which 158 DEGs were associated with heat tolerance.

In heat-stressed pregnant ewes, the molecular regulation of thermo-tolerance was revealed using blood transcriptomic analyses. The study identified a total of 358 DEGs and found stress induced non-viral immune mechanism involving apoptosis as a key molecular regulator of thermo-tolerance in ewes [104]. A study in heat-stressed pigs identified various patterns of DEGs in regulatory and effector tissues (muscle, adipose tissue, liver, blood, thyroid, pituitary, and adrenal glands), unravelling interactions between tissues related to oxidative stress upon heat exposure. The results of the study can thus aid in the comprehension of adaptation mechanisms during heat stress and in selection of pigs with high thermo-tolerance capacity [105].

Further transcriptome analysis can aid in understanding the molecular and metabolic responses of heat-stressed animals. On this line, a study in Holstein cows carried out liver transcriptomic analysis and identified 483 DEGs. The study results revealed the downregulation of all the protein coding genes in the mitochondria and the upregulation of six heat shock proteins in heat-stressed animals, indicating negative effects on mitochondrial integrity and metabolic homeostasis in liver. Furthermore, gene ontology (GO) disclosed the effects on protein folding pathway (upregulated) and oxidative phosphorylation (downregulated), suggesting compromised energy production as a result of mitochondrial dysfunction [106].

Whole transcriptome analysis has also been applied to assess the reproductive performance of animals during heat stress periods. Haire et al. [107] investigated the gene expression in multiple tissues (pituitary, ovary, and hepatic tissues) of heat-resistant Turpan black sheep compared to the heat-sensitive Kazakh sheep. They found 32, 49, and 69 genes to be upregulated and 39, 60 and 145 genes to be downregulated in pituitary, ovarian, and hepatic tissues, respectively, involved in the energy and ovarian steroidogenesis. They concluded that pituitary signals under heat stress would affect ovarian steroidogenesis and change hepatic energy metabolism, thereby reducing the reproductive potential in animals. A similar alteration in the oviductal cell’s gene expression was observed in dairy cows subjected to heat stress [108]. Furthermore, reports on investigating the impact of heat stress on the activation of the zygote genome in embryos using transcriptomic studies have also been studied. They observed a significant impact of high temperature on the activation of the zygote’s genome due to the alteration in the oxidative phosphorylation levels and mitochondrial function during heat stress conditions [109].

Li et al. [110] performed transcriptomic profiling of liver tissue in Hu sheep and revealed 520 (mRNA) and 22 (IncRNA) differentially expressed genes. The results helped to understand the underpinning molecular response during heat stress due to their strong associations with heat stress-related pathways, such as carbon metabolism, the PPAR signalling pathway, and vitamin digestion and absorption. Additionally, transcriptomic studies have helped in understanding the impact of heat stress on the rumen epithelium. They observed the upregulation of *HSP* expression during heat stress, ensuring the ruminal epithelium stays intact. These investigations help to understand the defence mechanism the body employs in heat stress situations such that suitable mitigation strategies can be developed [111].

Another study was conducted in crossbred cattle where the authors found 468 and 2273 upregulated and downregulated genes when exposed to chronic heat stress conditions. The upregulated DEGs were responsible for the development of neurones and sensory organs, the calcium signalling pathway, the Ras-proximate-1 signalling pathway, the mitogen-activated protein kinase (MAPK) and Smad signalling pathways, apoptosis, and oxidative stress. Likewise, the immune system, mRNA processing, B-cell receptor signalling route, nucleotide oligomerisation domain (NOD)-like receptors (NLRs) signalling pathway, and nonsense-mediated decay (NMD) pathway were the pathways where downregulated genes were highly expressed [112].

Furthermore, transcriptomic profiling can aid in the better understanding of heat stress-associated meat quality deterioration leading to production losses. A transcriptomic study in broilers suggested that the effects of chronic heat exposure on meat quality was related to signal transduction, immune system, transport and catabolism, cell growth and death, and muscle structure, in addition to altered metabolism and oxidative stress. An understanding of these intricate signal mechanisms can assist in identifying heat-tolerant birds [113]. A similar study assessing the transcriptomic profile of pectoralis muscle using RNA-Seq on heat-stressed chicken breeds (commercial broiler, Thai native chicken, and crossbred chicken) was conducted. They observed inflammatory responses to be more pronounced in broiler than the other breeds. The authors attributed the better adaptive mechanism in the two breeds to the actin cytoskeleton regulation and AMPK and MAPK signalling pathways [114]. Additionally, studying the skeletal muscle transcriptome also helped in understanding the molecular basis of maintaining body temperature in pigs at low temperatures [115].

A study in Brangus bulls was carried out to evaluate the skin transcriptome profiles during heat stress conditions and correlate them with between breed composition, phenotypic, and skin histological traits. The results revealed 4309 DEGs, 2113 downregulated and 2196 upregulated. Furthermore, in the same study, the enrichment and ontology analyses disclosed 132 GO terms and 67 pathways, including thermogenesis, glycolysis, gluconeogenesis, mitochondrial activity, antioxidant and immune response, and apoptosis, indicating simple passive and complex active heat-dissipation mechanisms [116]. Furthermore, in a comparative study between Kanni Aadu and Kodi Aadu goat breeds, the skin transcriptomics revealed Kodi Aadu goat breeds to have better climate-resilient potential as a greater number of DEGs were expressed in Kanni Aadu goats, indicating the latter to be evincing relatively higher stress response [117]. These studies are highly beneficial to understanding the thermo-tolerance of animals as these tissues (skin) undergo many molecular changes, as they are exposed first to climatic alterations. As a result, these studies have shown that RNA-Seq is an appropriate and promising technology for providing a better picture of the molecular mechanisms associated with thermoregulation.

### 6.3. Whole Metagenome Analysis

Metagenomics involves the study of the structure and function of complete nucleotide sequences that have been extracted and examined from every organism in a bulk sample (usually microbes). It refers to the application of sequencing techniques to analyse the totality of the genomic material present in a sample [118]. It is a technique that can identify and characterise organisms from various materials without regard to culture. Adopting techniques like 16S rRNA sequencing and whole genome shotgun metagenomics has made it possible to assess the structural and functional dynamics of microbial populations in a sample. The application of this NGS approach was extended to livestock samples like rumen liquor [119,120], skin [117], faeces [121], and so on to assess the alteration in the microbial population in response to various factors.

Numerous investigations have demonstrated that the host’s metabolic activities are impacted by the interactions between the rumen bacteria and the host [122]. Park et al. [123] observed alteration in the rumen microbiota (prokaryotes and protozoans) of lactating Holstein cattle when they were subjected to heat stress that in turn change the host’s metabolism. It was possible to link such alterations with several functional and metabolic pathways like energy production and conversion, defence mechanisms, lipid transport and metabolism, coenzyme transport and metabolism, cell cycle control, cell division, and more [119]. Employing metagenomics could also aid in understanding the differences in heat stress adaptive strategies between livestock breeds.

The comparative metagenomic study of the rumen microbiome between Holstein and Jersey cattle during heat stress aided in understanding the differences in thermal resilience between these cattle breeds. The study revealed Jersey cows to have better adaptive potential than Holstein cattle as more noticeable alterations in the rumen bacterial taxa (29) and functional gene abundance in Jersey cows were observed. This indicated that Jersey cows do not react highly to heat stress in hot weather, inferring that these modifications most likely aid in adaptation to climate change [119]. Similarly, Feng et al. [124] conducted an experiment on Holstein cows to explore the effects of heat stress on their microbial composition, function, and metabolism. They concluded that the young heifers were more susceptible to the alteration in the microbial community than the adult lactating cows. A similar metagenomic study investigating alteration in the rumen microbial population during heat stress was conducted in dairy calves. An increase in the butyrate-producing bacteria was observed as a response to heat stress in calves [125].

The diversity of microbes is much higher in buffaloes than cattle [126]. Yadav et al. [120] conducted a study in six buffalo heifers (Murrah) and observed no change in the dry matter intake, volatile fatty acid concentration, and digestibility during high ambient temperatures. However, in contrast, a metagenomic study of the rumen liquor revealed an increase in Firmicutes and a decreased abundance of Proteobacteria and Planctomycetes, along with eight new genera of microbes. The authors concluded that the rumen microbial population’s resilience triggered adaptive responses by varying their abundance to reduce the negative effects on fermentation and digestibility.

Metagenomic analysis of the ruminal fluid was also explored in small ruminants. A comparative study led by Sejian et al. [127] on three indigenous goat breeds (Osmanabadi, Salem black, and Malabari goats) revealed a considerable alteration in the rumen bacterial community at all taxonomic levels across control and heat-stressed groups of all three breeds. The authors observed Salem black to possess genetic superiority in altering the rumen microbial population which could impart them the potential to thrive well in harsh climatic conditions. Similarly, in another experiment, the impact of acute and chronic heat stress on the microbial population of rumen was assessed in dairy goats. The authors observed alteration in the microbial population during heat stress that led to a change in metabolism host and concluded that the production of dairy goats raised in harsh environments may be enhanced by the use of HS-resistant bacteria as probiotics to lessen the harmful effects of HS [128]. Further experiments studying the effect of heat stress on the diversity of microbes on goat skin was evaluated for the first time by Silpa et al. [117]. Their study revealed alteration in caprine skin microbiota due to heat stress.

Related studies were also performed in monogastric animals like pigs [129] and poultry [121]. Hu et al. [129] studied the intestinal microbial diversity and observed a decrease in *Lactobacillus johnsonii* and *Lactobacillus reuteri* and increased *Clostridium sensu* stricto microbes in heat-stressed pigs (Luchuan × Duroc). Changes in the concentration of short-chain fatty acids (SCFA) and biochemical indices were linked to these modifications. This led to intestinal mucosal damage and the activation of inflammatory signal pathways and immune response, thereby causing stress-induced inflammatory bowel disease in pigs. They also stated that pigs exposed to chronic heat stress exhibited altered microbes associated with gut immune function and depicted an increase in potential pathogens (e.g., Asteroleplasma, Shuttleworthia, and Mycoplasma) and suppression of beneficial bacteria (e.g., Coprococcus and Aeriscardovia), thereby inducing immune function disorder in heat-stressed pigs. In another study, Zhu et al. [121] performed a metagenomic analysis on faecal contents in heat-stressed laying hens and revealed taxonomic alterations in the gut microbiome. The *Firmicutes* were enriched in the control group at the phylum level, whereas their abundance declined following heat stress; *Bacteroidetes* showed a high enrichment (43%) in the faecal microbiota under heat stress, surpassing that of the layers at optimal temperature. They hypothesised that variations in the abundance of *Firmicutes* and *Bacteroidetes* could be linked to the reduction in feed intake in layers caused by heat stress, as well as the drop in triglyceride and cholesterol levels linked to obesity and liver disease.

It is clear from the research listed above that the microbial population may be impacted by climate change, which could worsen the harmful effects of heat stress on animals. Modifying or manipulating the composition of the ruminal microbiome may be a novel approach to lessen the effects of heat stress. Nevertheless, there is a dearth of information pertaining to the adoption of this methodology in assessing the impact of heat stress on livestock. Therefore, intensifying the application of this methodology to assess heat stress impact and responses in livestock and extending it to assess the effectiveness of amelioration strategies could embark a new journey towards heat stress mitigation.

### 6.4. Bisulfite Sequencing Analysis

Bisulfite sequencing analysis is the bisulfite conversion of genomic DNA combined with next-generation sequencing (BS-seq) to determine the methylation status of an entire genome, or the methylome, at the single-base resolution [130]. Unmethylated cytosines are preferentially deaminated when subjected to bisulfite ion treatment; these cytosines are then transformed to uracil through desulfonation. Mammals’ ability to regulate their gene expression largely depends on epigenetic processes, such as DNA methylation. Research on DNA methylation will advance our knowledge on the role that environmental variables play in the phenotypic variation in complex production and health traits. For a thorough analysis of DNA methylation, high-throughput sequencing is essential. Animal bioscience research has entered a new frontier using DNA methylation analysis. Through the process of mapping the DNA methylome, scientists can investigate an epigenetic mechanism that governs gene expression during heat stress related to production [131], adaptation [132], reproduction [133], and immunity responses [134] in animals and thereby aid in understanding the biological significance of changes seen as a response to heat stress in animals.

In 2024, Kumar et al. [133] reported differentially methylated cytosines in the promoter region of the genes encoding the channels responsible for Ca^2+^ exchange, *NPTN*, Ca^2+^ activated chloride channels, *ANO1*, and a few structure-related units such as septins (*SEPT4* and *SEPT6*), *SPATA*, etc., in the sperm cells of Murrah buffalo bulls. They suggested that the reduced semen quality in the Murrah buffalo bulls during the season-related heat stress may have been caused by promoter methylation in the genes controlling surface transporters and sperm structure. Similarly, a study to understand the seasonal impact of heat stress on bovine oocytes using transcriptomics and DNA methylation pattern study was conducted by Diaz et al. [135]. The authors observed several DEGs during summer season; however, they did not find any differences in their DNA methylation pattern and concluded that more advanced techniques like genome-wide bisulphite sequencing can give deeper insights to assess the DNA alteration at gene-specific loci [135].

Extending the application of this methodology to understand the association between heat stress and meat quality, Reith et al. [136] employed reduced representation bisulphite sequencing and assessed the impact of heat stress on the skeletal muscle of beef cattle. The observed DMRs were responsible for alterations in the oxidative stress activity, inflammatory response, and muscle growth that had resulted in reduced growth during thermal stress [136]. Similarly, Chen et al. [137] employed whole genome bisulphite sequencing technology to assess the DNA methylation of the *GNAS* region in response to heat stress. They suggested *GNAS* to be a potential gene that modulates heat stress as they observed an increase in methylation levels of the *GNAS* promoter region during heat stress.

Additionally, with this technology, it was possible to identify animals with climate-resilient potential and enhanced immune responses. Wang et al. [138] performed a study on Lvliang black goat by integrating transcriptome analysis and DNA methylation. Their results revealed 7833 differentially methylated regions and 102 differentially expressed genes that were associated with adaptation-related pathways (Lipid transport and immunity metabolism). Similar study investigating epigenetic variation in relation to the changing climatic conditions was conducted in sheep and goats [139]. Sajjanar et al. [140] performed whole genome bisulphite sequencing to compare the heat stress response for Hariana and Vrindavani cattle. They found varied genome-wide DNA methylation patterns between the two breeds. In comparison to the Vrindavani cattle, Hariana has 756 hypermethylated and 3845 hypomethylated CpGs, out of 4599 substantial differentially methylated CpGs. Additionally, the authors also found 79 genes related to cellular stress response processes to have exhibited both differential methylation and differential expression. They attributed it to be the reason for the difference in phenotypes between the two species and concluded that the epigenetic variations could be the possible reason for the Hariana cattle’s relative thermo-tolerance and long-term adaption to the tropical climate. In a similar study between indicine and crossbred cattle, in comparison to the crossbred cattle, zebu cattle that had acclimated to tropical climates expressed more stress genes, and they attributed that the expression of these genes could be regulated by DNA methylation [141].

In another study, Livernois et al. [134] conducted a DNA methylation study in the blood mononuclear cells of high- and low-immune responding Holstein cattle under heat stress. They identified several differential-mediated promoter regions associated with various biological processes, including immune function, stress response, apoptosis, and cell signalling. In conclusion, bisuphite sequencing technology provides newer insights to further understand the heat stress response in animals by studying their DNA methylation patterns. Furthermore, this type of study also revealed that the response of animals to heat stress varies among species [132], thereby helping to identify the most thermo-tolerant breed. Table 1 overviews pathways affected by heat stress in various livestock species using various biotechnological tools.

## 7. Statistical Tools to Establish Thermo-Tolerance in Livestock

### 7.1. Genome-Wide Association Studies (GWAS)

Genome-wide association studies (GWAS) examine hundreds of thousands of genetic variants across many genomes to identify those statistically linked to a particular characteristic or disease. The GWAS investigates associations between genotypes and phenotypes by comparing the allele frequencies of genetic variants in individuals with comparable ancestry but different characteristics. With the development of sophisticated statistical methods and the availability of greater density, the SNP chips can be used to identify more prevalent haplotypes among breeds. The results of GWAS can be used for various purposes, including understanding the underlying biology of a phenotype, calculating its heritability and genetic correlations, predicting clinical risks, guiding drug development initiatives, and deducing potential causal relationships between risk factors and health outcomes [156].

The capacity for adaptation or reduction in susceptibility to the harmful effects of high temperatures is known as thermo-tolerance in living organisms. Numerous species, including dairy cattle [144,148,157], sheep [142,158], goats [143], pigs [146,159] and poultry [147], have been shown to exhibit a genetic component linked to this favourable response to heat stress. Luna-Nevarez et al. [142] performed GWAS on pregnant ewes (Columbia × Rambouillet) and identified four candidate genes (*FBX011*, *PHC3*, *TSHR*, and *STAT1*) involved in HSP regulation. The authors identified four intragenic SNPs within these genes associated with thermo-tolerance, which can potentially be used as biomarkers in breeding programmes to improve the heat-tolerant potential of sheep. Aboul-Naga et al. [157] performed GWAS in Egyptian sheep breeds (Saidi, Wahati, and Barki) to identify candidate gene associated with adaptation during heat stress conditions. The authors identified SNPs linked to heat tolerance in the *RTN1*, *PRKG1*, *GSTCD*, and *MYO5A* genes related to endoplasmic reticulum stress, body thermoregulation, respiratory function, and melanin production. Since these genes are critical for the proper functioning of the biological system, this might account for the substantial correlation between them and heat tolerance. They also identified heat-tolerant genes (*STEAP3* and *GPAT2*) involved in lipid storage and fat deposition in sheep tails and muscle development (*KSR2*) to be strongly correlated with thermal stress. The authors attributed it to be a possible reason that the Egyptian sheep breeds outperform the temperate breeds in harsh climatic conditions [157]. A similar study was conducted by Kominakis et al. [158] to identify the genes responsible for the adaptation in the sheep breeds of Greece. The authors identified five candidate genes *TEX47*, *SRI*, *STEAP4*, *ZNF804B*, and *ADAM22* to have a substantial role in the adaptation process of these animal.

The GWAS have also assisted in improving the milk production in animals. Zidi et al. [143] identified genes that influence milk yield, milk composition, and cell death levels in the mammary glands of Spanish Florida Dairy goats. The authors detected heat shock 27 kDa-associated protein 1 (*HSPBAP1*) mapped to a considerable SNP (snp39045-scaffold-3419), which inhibits the anti-apoptotic and thermo-tolerance function of heat shock protein 27 kDa (*HSP27*) at the cell level. They also identified other genes involved in milk composition, namely, kappa casein (*CSN3*), acetyl-coenzyme A carboxylase alpha (*ACACA*), and malic enzyme 1 (*ME1*). In a similar GWA study conducted in dairy cows, for the first time, candidate genes were identified to improve the milk production traits in dairy cattle raised in tropical conditions [160]. Furthermore, Macciota et al. [144] reported a reduction in milk production as the temperature–humidity index (THI) increased in Italian Holstein cattle under heat stress. The authors observed a substantial correlation between two and four SNPs with the slope of milk yield and fat percentage level and two correlations with the level and slope of protein percentage, respectively, which were associated with the mechanisms of heat tolerance.

GWAS have been widely carried out in pigs to explore their thermo-tolerance potential. Kim et al. [145] used porcine high-density single SNP bead chips to find substantial gene region associations for rectal temperature on *SSC12*, respiration rate on *SSC14* and *SSC16*, as well as feed efficiency and weight loss on *SSC13* in crossbred pigs. A similar study was conducted by the same author in gilts and identified several candidate genes. The authors linked regulation of cellular stress response to genes associated with respiration rate (*PAIP1*, *NNT*, and *TEAD4*), rectal temperature (*LIMS2*, *TTR*, and *TEAD4*), and skin temperature (*ERBB4*, *FKBP1B*, *NFATC2*, and *ATP9A*) that can be used to understand the heat stress response in pigs [146]. Further studies were also carried out in pigs to understand the underlying mechanism that causes the reduction in feed intake during heat stress that affects the growth of the animals [159].

Using GWAS, Otto et al. [148] identified candidate genes (*TXNRD2*, *LIF*, *DGCR8*, and *OSM*) associated with rectal temperature in Gir × Holstein crossbred animals. They hypothesised that alleles specific to the Holstein breed might be linked to a more intricate reaction to the effects of heat stress, as these animals require a complex genetic architecture to protect the body from the harmful effects of heat stress. This may contribute to Holstein animals being more susceptible to heat stress than Gir animals.

Van Goor et al. [147] identified quantitative trait loci associated with body temperature (BT), body weight (BW), breast yield, and digestibility in an intercross line of chickens during heat stress to improve the selection criteria during breeding. They identified 4, 11, 3, and 3 QTL for body temperature, body weight, dry matter digestibility, and breast muscle yield, respectively, and found the data to aid genomic selection in the breeding of heat-tolerant chickens. Additionally, GWAS are also conducted nowadays by linking disease prevalence to heat stress [161]. Such efforts will provide deeper insights and help in developing animals that can withstand the harsh conditions with disease resistance potential. Therefore, GWAS prove to be a promising biotechnological tool, and selecting animals adapted to climate change could be a way to guarantee sustainable livestock production in the changing climatic scenario.

### 7.2. Applications of Selection Signatures

Selection signatures are the distinctive genetic patterns or traces that are left behind in the genomic regions that are under selection. The patterns of variation among selected loci and in neutral loci connected to them tend to change in particular ways due to selection. Selection signatures are the genomic traces left by selection that can be used to identify loci under selection [162]. It provides knowledge emerging from a fundamental understanding of the evolutionary processes that shapes genomes, providing functional knowledge about genes and genomic regions. Therefore, the identification of selection signatures is currently one of the main priorities of evolutionary geneticists. It may also help identify genes associated with ecological traits that are challenging to identify through laboratory experiments, such as genes related to tropical adaptation. This methodology aids in finding the underlying mutations that give a particular population or species a selection advantage [163]. To find selection signatures, several statistical techniques based on reduced local variability, site frequency spectrum, linkage disequilibrium, and population differentiation have been developed [162]. These approaches have been used in livestock to detect potential loci and candidate genes that have undergone positive selection and influence complex traits. These studies have been very helpful in the ongoing global warming crisis in livestock as they aid in identifying the genomic regions that are responsible for adaptation to hot climates. Several experiments have been conducted in this aspect and Peng et al. [164] reported it in goat breeds and identified genomic regions linked to adaptation traits to both hot and cold climatic conditions.

Guo et al. [149] studied 59 whole genomes of native chickens from temperate and tropical regions of Northern China and South Asia to determine the genes contributing to heat tolerance for a direct breeding programme. Using nucleotide diversity (θπ) and F_ST_ statistical measurement tools, they identified 34 genes with a positive selection signature in chickens raised in tropical areas that were possibly found to be associated with adaptation to the hot climate. Among them, they found variation in the thyroid stimulating hormone receptor gene (*TSHR*) that may control a chicken’s metabolic rate to improve their heat tolerance and help them adapt to high ambient temperatures in tropical regions that can be used as a candidate gene in breeding programmes to develop heat-tolerant chickens. A similar study was conducted by Asadollahpour Nanaei et al. [150] between Iranian indigenous and commercial line chickens. The authors identified several immune responses and HSP-related genes (*HSP70*, *HSPA9*, *HSPH1*, *HSP90AB1*, and *PLCB4*) that have undergone positive selection in indigenous breeds associated with adaptation mechanisms in hot environments. The F_ST_ method measurements were carried out in Iranian buffalo breeds, Azeri, and Khusteni, while performing their genomic scan to identify signatures of positive selection associated with the adaptation to different environments [155]. Genotyping was performed using the Axiom^®^ Buffalo Genotyping 90 K Array. On comparing the data of the indigenous buffalo with that of *Bos taurus*, the authors identified putative candidate genes under selection, namely, *NDFIP1*, *ACTR3*, *ARHGAP26*, *BOLA-DQB*, *CLN8*, *MYOM2*, *FBX09*, *SERPINF2*, and *BOLA-DRB3* associated with physiological pathways including milk production, cytoskeleton organisation, growth, metabolic function, and others [155]. A similar selection signature study using Fst and hapFLK tests were conducted in Iranian sheep breeds. They identified several genomic regions to be under selection in the sheep breeds adapted to the hot climate [165].

Researchers discovered that African cow breeds subjected to positive selection have genes linked to their heat tolerance mechanisms [152]. They analysed the genomes of five native African cattle breeds and four commercial breeds using cross-population composite likelihood ratio (XP-CLR) and cross-population extended haplotype homozygosity (XP-EHH) statistical techniques. They showed that 296 (XP-EHH) and 327 (XP-CLR) positively selected genes are involved in the development of sweat glands and perspiration, the oxidative stress response, the osmotic stress response, the heat shock response, hair and skin characteristics, feed intake and metabolism, and reproductive functions. They concluded that the identified genes and pathways facilitate the enhanced heat tolerance mechanism in African cattle, either directly or indirectly. Using population statistics methods like F_st_, XP-CLR, and XP-EHH, Li et al. [153] conducted a similar study and identified selection signatures involving various pathways (sweating, calcium signalling pathway, heat shock, oxidative stress response, coat colour, feed intake, and reproduction) associated with thermo-tolerance and disease resistance in Dehong humped cattle when comparing against the genomes of Diqing and Zhaotong cattle breeds. In another study, Zhang et al. [166] calculated the genome-wide weighted FST and nucleotide diversity in the native goat breeds of Pakistan and identified genes under positive selection that were related to milk production and adaptation traits in the tropical climate. The authors identified a number of potential genes linked to immunological response, milk production features, and adaptation to the local environment. Similarly, in the study conducted by Saravanan et al. [167], sheep populations revealed genomic regions under selection that are linked to adaptation pathways to hot climates such as cytokine receptor binding and keratin filament organisation.

In the coastal zone of the Western Desert in Egypt, Caprine and Ovine genotype data were analysed by Kim et al. [154] for Barki goats and sheep, which are native to a hot, dry climate. Using genome-wide SNP scans, selection signatures covering multiple genes that impacted traits for adaptation to hot and dry environments, including energy and digestive metabolism (*MYH*, *ALDH1A3*, and *TRHDE*), body size and development (*BMP2*, *BMP4*, *GJB2*, and *GJA3*), nervous system and autoimmune response (*IL2*, *IL7*, *IL21*, *IL1R1*, and *GRIA1*), and thermo-tolerance (melanogenesis) (*FGF2*, *PLCB1*, and *GNAI3*) were identified. Together with a shared selection signature spanning a conserved syntenic segment to bovine chromosome 12 on Caprine and Ovine chromosomes 12 and 10, respectively, they found eight common candidate genes under selection in the two species. A similar study was conducted on eight indigenous cattle breeds of India to identify the genes that have undergone selection related to the adaptation traits. They employed bovine 50K chip data and identified several genes in the selection signature areas, namely, *CLPB*, *HSPA1B*, *HSP90AB1*, *HSP20*, *HSPB2*, *HSPB3*, *HSF4*, *GAP43*, *MITF*, and *MCHR1* that were responsible for thermo-tolerance and disease resistance [168].

There are also related studies reported to identify the selection signatures for adaptation in pigs. Wang et al. [151] performed a comparative genomic analysis between Xiang pigs and Meishan, Duroc, and Landrace pigs. They identified genes (*PGDE1A*, *EXPH5*, *VEGFC*, *SDR9C7*, *UVSSA*, *NR2E1*, *SERPINB10*, *MYO1A*, *SERPINB8*, and *SLC2647*) involved in maintaining the mechanical stability of skin, behavioural defence response, response to ultraviolet (UV) radiation, UV-induced cellular response, and regulating oxygen homeostasis that were associated with adaptation in the selected regions of Xiang pigs.

**Figure 1:**
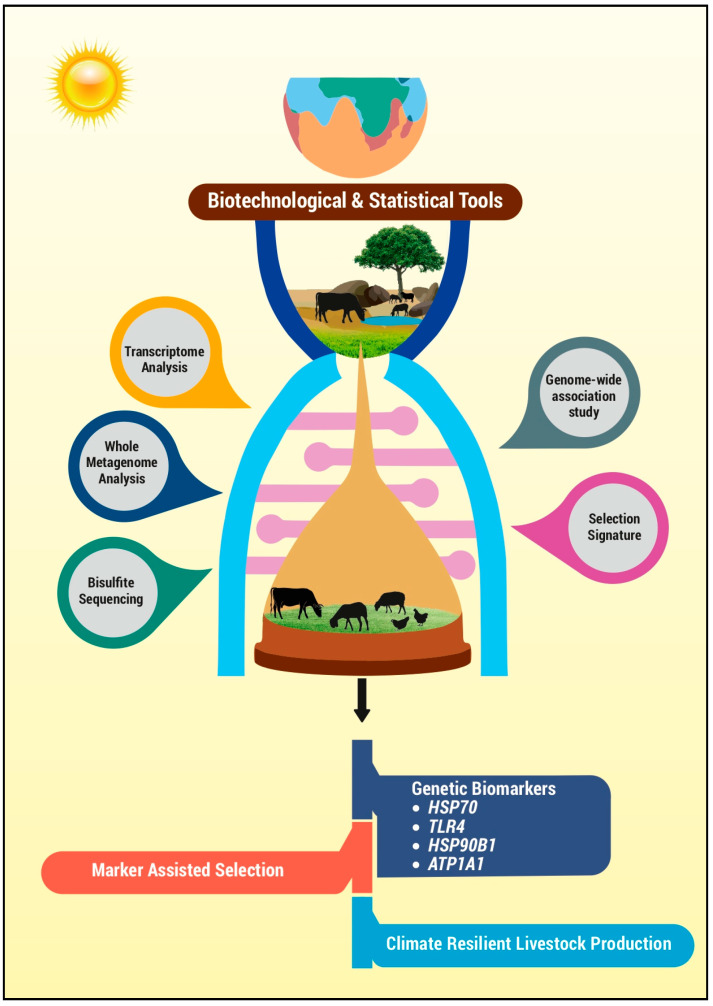
Applications of biotechnological and statistical tools to establish climate-resilient livestock production.

Thus, analyses of signatures selection provide a comprehensive picture of the adaptation of different species to the hot environment and help identify the possible genes associated with thermo-tolerance that can be used as biomarkers during breeding programmes to develop climate-resilient animals. Table 1 overviews pathways affected by heat stress in various livestock species using various statistical tools. Figure 1 describes the various biotechnological and statistical tools for establishing climate resilience in livestock.

## 8. Genomic Selection to Mitigate the Effects of Heat Stress

The selection of superior adapted breeds that can successfully endure the heat stress challenges may be aided by the identification of certain genes and gene markers that are linked to thermo-tolerance with the help of genomics [3]. Genomic selection is a type of marker assisted selection (MAS) that includes the use of gene markers, such as microsatellites, single nucleotide polymorphisms (SNPs), SNP chips, genome-wide association studies (GWAS), sequencing, and other related technologies [169]. Genomic selection in breeding programme has the potential to improve the precision and efficacy of conventional breeding and advanced breeding techniques.

The field of genomics has made it possible to pinpoint and incorporate major genes (QTLs) linked to thermo-tolerant traits related to adaptation [170], reproduction [171], and productivity [172]. Fathoni et al. [171] reviewed various genetic techniques including conventional method, marker-assisted selection, and genomic selection in crossbred Thai–Holstein cattle to find the most appropriate approach for improving the reproductive traits under thermal stress conditions. The authors concluded genomic selection to be the most potential tool that yields remarkable genetic gain and single-step genomic BLUP (ssGBLUP) method as the ideal approach for the less heritable reproductive traits under heat-stressed conditions. Similarly, Garner et al. [172] conducted a study in Holstein cattle under controlled climate chambers to highlight the impact of genomic predictions as a tool improve the thermo-tolerance potential in animals. They found animals identified via genomic breeding values as heat tolerant and exhibited a lower decline in milk production and a lower rise in core body temperature compared to cows identified as heat sensitive. These studies suggest that genomic selection is an ideal method to mitigate the effects of heat stress by boosting the robustness, welfare, and productivity of the animals in the changing climatic conditions.

Additionally, genomics combined with other omics technologies, such as transcriptomics, proteomics, and metabolomics, could provide a more holistic view of the molecular mechanisms governing thermo-tolerant traits [173]. Hence, genomic applications can be widely used for the genetic improvement of animals that result in the identification and development of more climate-resilient breeds to improve the livestock productivity, thereby benefiting the livestock sector. This will help in expanding the knowledge to understand the molecular mechanism underpinning heat stress in livestock breeding programmes that offers a new perspective for future research.

## 9. Applications of Marker-Assisted Selection

Marker-assisted selection (MAS) involves the inclusion of genomic data with phenotypic data to improve the selection response to the conventional approach [11]. It is an indirect selection approach where genetic markers are employed to identify the presence of desirable genes [174]. The trait of interest is not selected directly but rather by a marker linked to it. The goal of this approach is to improve genetic evaluation and selection by combining all genetic data from markers and quantitative trait locus (QTL) with phenotypic data [174].

Successful application of MAS in breeding programmes requires advances in gene mapping, marker genotyping, QTL detection, genetic evaluation, and the development of breeding strategies and programmes for using molecular genetic information in selection and mating programmes [175]. The MAS has a wide range of applications and can be used to find genes associated with genetic disorders [176], improve meat quality [177], thermo-tolerance [178], improve immunity [179], improve milk production [69], and improve disease identification [180].

Livestock thermo-tolerance is regarded as a quantitative trait efficiently regulated by the genomic regions located at the target genes. The selection of superior adapted breeds that can effectively withstand the challenges of heat stress may be aided by identifying genes and gene markers related to thermo-tolerance. The P14 locus within the bovine *ATP1A1* gene has been identified as a DNA marker for bovine heat tolerance via marker-assisted selection, and studies indicate that the bovine blood *ATP1A1* gene may be able to lessen the effects of heat stress in Tharparkar and Vrindavani cattle [181]. There have been many QTL studies in chickens covering a wide range of traits including growth, meat quality, egg production, disease resistance, and behaviour [182].

If the genes controlling resistance to a specific disease were identified, it would be possible to transfer them from the indigenous breed into the improved breed, thus, producing stock that has an increased production potential and is resistant to endemic disease. The introgression of disease-resistant genes into the improved breeds would be achieved initially by crossing the indigenous and improved breeds [11]. For example, Holstein cows in Florida were bred for the dominant “slick” gene, which causes extremely short hair growth after it was originally discovered in the Senepol breed of beef cattle in the Virgin Islands. They found the resulting offspring to regulate their body temperature better during heat stress than cows with normal hair [183]. Therefore, through molecular marker technology, it is possible to transfer heat-resistant genes from indigenous breeds to temperate breeds to simultaneously improve their productivity, disease tolerance, and heat tolerance.

The use of molecular markers for improving the meat quality or growth traits of beef cattle is important for beef breeders. Marker-assisted selection using SNPs identified in genes, such as *LPL*, *CRTC2*, *SIRT1*, *SIRT2*, *SIX4*, *ZBTB38*, and *UCPs* as selection criteria for body measurements and meat traits in beef cattle, has been reported to be beneficial in selection and breeding programmes [177]. Similarly, the identification of several genes associated with body measurement and meat traits in beef cattle, with a strong focus on single genes and mapping QTLs to make them available soon, can be highly beneficial for farmers.

The presence of the gene responsible for the resistance against a certain disease can be checked through molecular markers selection criteria. Griesbeck-Zilch et al. [179] investigated the genetic and molecular mechanisms of a QTL on *Bos taurus* chromosome 18 that affected udder health, as well as the resistance to mastitis. The authors observed the mRNA expression of toll-like receptor 2, tumour necrosis factor-α, IL-1β, IL-6, IL-8, regulated upon activation, normal t-cell expressed and secreted (RANTES), complement factor C3, and lactoferrin to be substantially elevated in cells isolated from less-susceptible animals in the marker-assisted selection groups. Hence, these observations provided the first insights into genetically determined divergent reactions to pathogens in the bovine mammary gland and indicated that the application of QTL information could be a successful tool for the selection of animals resistant to mastitis.

Yodklaew et al. [184] conducted a GWAS for milk characteristics in a Thai multi-breed population and identified 366 markers substantially associated with these traits. However, these markers, being influenced by environmental temperature, may not be effective markers when considering another population. Hence, it is necessary to consider the climatic conditions in a particular region when incorporating breeding strategies. Therefore, genomic approaches aid in the characterisation, identification, and conservation of heat-tolerant livestock breeds, which are the characteristics of the future challenging climate. By combining genomic selection and phenotypic selection, we can accelerate the breeding of highly productive and heat-tolerant livestock breeds. Further research should focus on the molecular characterisation and identification of indigenous breeds and on the identification of genes/genomic regions associated with thermoregulation, feeding, and production efficiency to develop suitable adaptive and mitigation strategies.

## 10. Different Genetic Biomarkers for Thermo-Tolerance in Livestock

Genetic markers are essential for linkage and association studies; they are defined as specific DNA sequences with a known location on a chromosome [185]. The biomarkers are hereditary variations that are stable, quantifiable, or detectable using an appropriate technique that can be used to detect the presence of a particular genotype or phenotype. With the ability to determine an animal’s genetic composition and thereby predict its behaviour, molecular markers hold promise for use in animal breeding [186]. These genetic biomarkers serve as a potential tool for establishing heat-tolerant potential in animals. In marker-assisted and genome-wide selection applications that aim to develop heat-tolerant breeds, the heat shock genes associated with thermo-tolerance serve as potent biomarkers [66]. Various reports have identified potential candidate genes associated with growth [187], production [188], reproduction [83,189,190], adaptation, and immune response [190,191] during heat stress that can serve as biomarkers in breeding programmes.

Various biomarkers have been reported to improve the reproductive efficiency in animals subjected to heat stress. Molecular markers (*FBXO11*, *PAM*, and *STAT1*) have been identified by Castilo-Salas et al. [189] to improve the reproductive efficiency of sheep (Pelibuey ewes) raised under heat stress conditions. Contreraz-Mendez et al. [190] reported candidate genome-wide SNPs on the genes *AMH*, *IGFBP1*, *LRG5*, and *TLR4* associated with anti-mullerian hormone (AMH) to have plausible roles related to fertility and thermo-tolerant traits in Holstein cows that can serve as biomarkers to improve the reproductive performances in animals exposed to heat stress. Similarly, Said and Putra [83] detected 2 SNPs (g.1117G/A and g.1125A/C) in the 5′ UTR region of *HSP 70* in Pasundan cattle that improve the service per conception rate, thereby increasing their thermo-tolerance capacity. Additionally, during the hot season, the semen quality of pigs (Duroc, landrace, and Yorkshire) was found to be closely associated with five SNP sites found in the 5′ flanking region of the *HSP 70.2* gene by Huang et al. [82]. Furthermore, using transcriptomic studies, Ozmen et al. [191] identified candidate genes (*COPS5*, *HSPA8*, *PSMC6*, *POLR2L*, and *TPI1*) during their study on heat-stressed bovine oocytes that can help reduce the impact of high temperature on oocytes.

Hooper et al. [192] identified the expression of *HSP70* and *ACTHR* genes on peripheral blood mononuclear cells in chronic heat-stressed Saanen goats that can be used as biomarkers for studying the long-term effects of heat stress.

Luo et al. [193] identified QTL regions linked to the rectal temperature (RT), respiration rate score (RS), and drooling score (DS), three physiological markers of the heat stress response in Holstein cattle. In their study, 2627 genes showed considerable upregulation, while 369 showed substantial downregulation according to RNA-seq analyses in the heat-stressed cattle. By combining the weighted single-step genome-wide association study (WssGWAS) and RNA-seq data, they identified primary candidate genes linked to physiological markers of heat stress in Holstein cattle, namely, *PMAIP1*, *SBK1*, *TMEM33*, *GATB*, *CHORDC1*, *RTN4IP1*, and *BTBD7.* A similar RNA-seq study on the CircRNA expression during heat stress revealed potential genes like *H1F1A*, *NR3C1*, and *ROCK1* in Holstein cows to develop heat-tolerant animals that can withstand the hot climatic conditions [194].

Using PCR–DNA sequencing, the molecular characteristics of the growth and heat tolerance genes in lambs from Barki and Aboudeleik were determined [187]. The authors identified SNPs in the genes related to growth and heat tolerance and identified biomarkers for sheep growth performance, namely, *CAST*, *LEP*, *MYLK4*, *MEF2B*, *STAT5A*, and *TRPV1*. Likewise, a GWAS study was conducted to identify genes linked to thermo-tolerance characteristics and milk production in Holstein cows raised in warm, humid conditions [188]. The authors reported that under heat stress, the molecular mechanism that controls milk production in cows appears to be influenced by SNPs in the genes *TLR4*, *GRM8*, and *SMAD3*.

Habashy et al. [195] found that DNA methylation of TXNRD and Prdx1 can be utilised as biomarkers of oxidative damage in meat-type chickens (Cobb500) under chronic heat stress while assessing the acute and long-term effects of heat stress on the thioredoxin–peroxiredoxin system’s gene expression and DNA methylation. Similarly, Karami et al. [196] identified *ATP9A* as a potential gene by employing reduced representation bisulphite sequencing on their chicken’s embryo.

Liu et al. [197] created a high-resolution map of selection signatures in the Shanghai Holstein cattle population through two complementary analyses based on GGRS data, expanding the range of selective signals in the cattle genome. The study’s selective signals strongly suggested Shanghai’s greater acclimation to hot, humid conditions. Some candidate genes, including *ITGA9*, *ACAT2*, and *PLAC8*, were found in potentially selected regions, and the variations in adaptation to particular environments and production systems were explained. A similar selection signature study in the tropical Abigar cattle was conducted using whole genome resequencing technology and *DNAJC18*, *HOXC13*, and *RXFP2* were identified as potential genes associated with cattle adaptation to the hot climatic conditions [198]. Van Goor et al. [199] performed GWAS for all physiological alterations in chicken blood under heat stress. They identified 61 QTL, which are found on GGA (*Gallus gallus* chromosomes) 1, 3, 6, 9, 10, 12–14, 17, 18, 21–28, and Z. The angiopoietin pathway was substantially enriched based on functional analysis of the genes in these QTL regions. They detected that the mapped QTLs could be used as markers for genomic selection to improve poultry’s resistance to heat. Barreto Sanchez et al. [200], in a liver transcriptomic study, revealed that Guang Ming chickens’ *HSP90B1* and *HSPA5* and Beijing You chickens’ *CPT1A* and *ANGPTL4* may be potential biomarkers of heat stress in poultry.

Furthermore, as stated in the earlier sections, heat stress-related physiological alterations may affect the microbiome’s makeup. Researchers have stated that some of the substantially altered microbes can be used as biomarkers. For instance, Czech et al. [201] studied the different genera that are associated with the respiratory score, drooling score, and rectal temperature during heat stress. Using a faecal 16S rRNA gene sequencing method, they identified 12 phyla and a total of 24 genera that were associated with heat stress metrics in Holstein cows. Among them, *Acidobacteria* and *Gemmatimonadetes* were observed to be the most substantial phyla, while the most substantial genera were stated to be *Rhizobium* and *Pseudobutyrivibrio*. The researchers concluded that the substantially altered bacteria may be strongly linked to heat stress and could be utilised as biomarkers in future microbiological research.

Dangi et al. [202], reported *HSP70* to be the most sensitive to temperature fluctuation and stated its potential to be utilised as a considerable molecular biomarker for heat stress in Barbari goats. In recent days, a comprehensive multi-omics study was performed in Holstein cattle to better understand the molecular mechanism that helps the animal maintain homeostasis during high temperature [203]. Such advancements can therefore aid in identifying candidate genes that can be used as a biomarkers in MAS to develop thermo-tolerant breeds. Such advancements may provide a possibility of finding a permanent solution to an animal’s adaptability to the changing climate scenario. Table 2 and Table 3 depicts the various identified biomarkers related to adaptation and production traits associated with heat stress in livestock, respectively.

## 11. Future of Genomic Research: Advancements Towards Third Generation Sequencing

Since its advent, DNA sequencing has progressed through several stages of refinement and development. The first methods to sequence DNA dated back to the 1970s, with chemical sequencing by Maxam and Gilbert [204] and the chain termination method by Sanger and colleagues [205] being the most prominent. Refinements, improvements, modification, and commercialization aided towards progression to the second generation, also known as next-generation sequencing methods [206]. Most genomic studies in livestock employ the NGS methods. Furthermore, yet another development was the establishment of third generation sequencing (TGS) which is stated to provide significant advantages over NGS due to its methodology that involves sequencing of native DNA directly without amplification [207]. The potential of TGS methods, like the Oxford nanopore technology (ONT) and single-molecule real-time sequencing (SMRT), to offer long read length, relatively cheaper sequencing cost, and easy sample preparation are much appreciated. Though in the developing stage, there are scant reports on the use of TGS on livestock [208,209].

At present, TGS technology in livestock genomics have mainly been applied for studies associated with genome assembly, structural variation detections, transcriptome sequencing, and epigenetic analysis [210]. This methodology however is also reported to incur higher error rates when compared to NGS methods. However, with focus on optimising sequencing functionalities and controlling the cost of sequencing, TGS has the potential to be a routine technology in livestock breeding. TGS is proposed to be essential in the discovery of rare genes by conducting population-based genetic breeding and conducting studies on single-cell whole transcriptome and epigenetics [210].

## 12. Climate Change on the Re-Emergence of Diseases and Their Related Genetic Resistance

The environmental changes as a result of fluctuating climatic factors have a catastrophic effect on disease distribution dynamics. The major indirect detrimental effects of the changing climate on livestock health and production are suggested to be due to microbial load, vector-borne disease distribution pattern, feed scarcity, and food-borne diseases [14]. For instance, the changing wind pattern affects the dispersal of flies like mosquitoes, sandflies (*Phlebotominae*), and midges (*Culicoides*). In addition, the climate change-associated global warming more likely causes an alteration in disease distribution, and the marginal zones becomes high-risk spots due to increased vectors in these areas [211]. The lengthened summers increase or decrease the infection cycles; for example, arthropod vector-borne disease cycle is established to extend in warm conditions. Furthermore, food scarcity leads to stress, immune dysfunction, and improved predisposal of livestock animals to a wide range of disease [212].

Introduction of novel pathogens is possible due to altered migration patterns of animals in search of food and water. It is also established that climate change triggers ecological invasion, bringing in alteration in sorting process that leads to genetic adjustments of the novel disease agents. This phenomenon consequently leads to the emergence of new infectious diseases [212]. In addition to the introduction of new diseases in recent years, different vector-borne and zoonotic diseases have been reported to re-emerge as a result of climate change. For instance, listeriosis, leptospirosis, rift valley fever, and yellow fever have been considered to be some of the major emerging zoonotic diseases as a result of climate change [212]. The most frequently addressed climate change-associated emerging and re-emerging diseases include bluetongue viral disease, rift valley fever West Nile fever, African horse sickness, lumpy skin disease, leishmaniasis, epizootic haemorrhagic disease, tick-borne disease, parasitic disease, pasteurellosis, avian influenza, anthrax, blackleg, and rabies [213].

Breeding for disease resistance is gaining a lot of significance among the livestock breeders. This approach was understood to have the added advantage of improving the productivity, health, and welfare of animals [214]. Resistance to disease is a threshold trait with low heritability making its selection and breeding a cumbersome approach. Selective breeding and crossbreeding were the initial approaches considered for improving resistance against diseases [214]. With the advancing years, researchers have identified candidate genes and markers associated with disease resistance using GWAS and quantitative trait loci mapping in livestock [215]. Apart from the molecular tools, gene-editing technology was also reported to hold great potential in breeding for disease resistance in livestock. This approach can provide sustainable development to the livestock industry [216].

## 13. Conclusions

Thermo-tolerance is a heritable variable wherein, through genetic selection, it is possible to improve tolerance to heat stress provided appropriate phenotypic information and tools are available. The advancements in NGS, bioinformatics, and statistical analysis have paved the way to obtain quicker access to the molecular mechanisms associated with thermo-tolerance in farm animals. However, compared to other economic traits (e.g., milk production, meat production, reproduction, etc.), relatively fewer researchers have adopted the NGS approaches in heat stress and adaptation studies in livestock. The documented reports on the usage of NGS approaches, like whole genome sequencing, whole transcriptomics, metagenomics, epigenetics, and so on, in heat stress studies in livestock have revealed several candidate biomarkers. Most of these identified biomarkers were stated to have a potential impact on identifying thermo-tolerant/susceptible livestock breeds/species. It is therefore necessary to target multi-institutional and multi-disciplinary approaches to conduct further research in this line that can eventually aid in the breeding of thermo-tolerant farm animals.

## 14. Future Perspectives

Recent developments in molecular genetics through the generation of several biotechnological and statistical tools have helped to identify advanced biomarkers to produce more heat-tolerant breeds that can optimally withstand extreme climate conditions (Figure 1). Although there have been several reports identifying such biomarkers through the NGS technologies, more efforts must be made to intensify their applications. One such option would be incorporating the identified biomarkers into the existing breeding programmes. Most of the conventional breeding policies primarily focuses on improving production traits. However, this must be redefined by incorporating adaptation and low methane emission traits. Additionally, a multidisciplinary approach of integrating other omics technologies like proteomics and metabolomics with these NGS tools can aid in obtaining a better understanding of the mechanisms and pathways involved in heat stress response and adaptation in farm animals. The quest towards the development of newer and robust DNA-sequencing technologies resulted in the establishment of third generation sequencing (TGS) technologies. This has found widespread application in human and livestock genetics concerning studies on genomic, transcriptomic, and epigenetic research. TGS has not been extensively adopted unlike NGS in assessing genetic potential of livestock and/or their thermo-tolerant potential. The TGS may therefore be proposed to play a pivotal role in strengthening future omics research towards heat-tolerant livestock. The policymakers must also consider the agroecological conditions of the region rather than framing a generalised breeding scheme, as differences in the adaptation potential of livestock breeds based on the agroecological zones have also been reported. Furthermore, a robust approach comprising these omics tools along with innovative technologies like artificial intelligence and machine learning could help towards the development of climate-smart animal agriculture and ensure future sustainable livestock farming.

## Figures and Tables

**Table 1:** Overview of pathways affected by heat stress in livestock species using various biotechnological and statistical tools.

Methodology	Species	Breed	Targeted Trait	Targeted Tissue	Pathways Affected	Reference
Transcriptomic analysis	Cattle	Holstein	Milk production	Mammary gland	Downregulation of milk protein-encoding genes, protein synthesis and amino acid and glucose transport upregulation of immune function and inflammation.	[100]
	Buffalo	Riverine buffalo	Milk production	Mammary epithelial cells	Cellular apoptosis and necrosis along with an overall decrease in MEC cell viability and proliferation.	[102]
	Buffalo	Nili ravi × Murrah	Thermo-tolerance/adaptation	Blood	Cytokine–cytokine receptors interaction pathway.	[103]
	Sheep	Turpan black sheep	Reproduction	Pituitary, ovary and hepatic tissues	Ovarian steroidogenesis and hepatic energy metabolism	[107]
	Sheep	Hu sheep	Adaptation	Liver	Heat stress-related pathways, carbon metabolism, the PPAR signalling pathway, and vitamin digestion and absorption.	[110]
Bisulfite sequencing	Buffalo	Murrah	Reproduction	Sperm cells	Methylation in the genes controlling surface transporters and sperm structure.	[133]
	Goat	Lvliang black	Adaptation	Blood	Lipid transport and immunity metabolism.	[138]
	Pigs		Meat production/growth	Longissimus dorsi muscle	Energy and lipid metabolism, cellular defence and stress responses, and calcium signalling pathway.	[131]
	Cattle	Holstein	Immune response	Blood mononuclear cells	Immune function, stress response, apoptosis, and cell signalling.	[134]
GWAS	Sheep	Columbia–Rambouillet crossbred ewes	Adaptation		Regulation of heat shock proteins.	[142]
	Goat	Spanish Florida Dairy goats	Milk production		Inhibits the antiapoptotic and thermo-tolerance function of HSP27.	[143]
	Cattle	Italian Holstein	Milk production		Milk yield, fat and protein percentage.	[144]
	Pig	Crossbred pigs	Adaptation		rectal temperature, respiration rate, feed intake, and body weight loss.	[145]
	Pig	Crossbred gilts	Adaptation		Skin temperature, rectal temperature, and respiration rate.	[146]
	Poultry	Intercross line	growth		Body temperature, body weight, breast yield, and digestibility.	[147]
	Cattle	Gir × Holstein	Adaptation		Rectal temperature.	[148]
Selection Signature	Poultry	Native chickens from temperate and tropical regions of Northern China and South Asia	Adaptation		TSHR control chicken’s metabolic rates to improve their heat tolerance.	[149]
	Poultry	Iranian indigenous chicken	Immune response and adaptation		Immune responses and HSP-related genes undergone positive selection.	[150]
	Pig	Xiang pigs	Adaptation		Behavioural defence response, maintaining the mechanical stability of skin, UV-induced cellular response, response to ultra violet (UV) radiation, and regulating oxygen homeostasis.	[151]
	Cattle	African cattle	Adaptation		Development of sweat glands and perspiration, the oxidative stress response, the osmotic stress response, the heat shock response, hair and skin characteristics, feed intake and metabolism, and reproductive functions.	[152]
	Cattle	Dehong humped cattle	Adaptation		Heat sweating (calcium signalling pathway), heat shock, oxidative stress response, coat colour, feed intake, and reproduction.	[153]
	Sheep	Barki sheep	Adaptation		Energy and digestive metabolism, body size and development, nervous system and autoimmune response, and thermo-tolerance.	[154]
	Goat	Barki goat	Adaptation		Energy and digestive metabolism, body size and development, nervous system and autoimmune response, and thermo-tolerance.	[154]
	Buffalo	Iranian buffalo: Azeri and Khusteni	Adaptation		Milk production, cytoskeleton organisation, growth, metabolic function.	[155]

GWAS: genome-wide association studies.

**Table 2:** Overview of identified biomarkers related to adaptation traits associated with heat stress.

Traits	Species	Breed	Methodology	Biomarkers	Reference
Adaptation	Goat	Saanen	Gene expression	*HSP70* and *ACTHR*	[192]
Adaptation	Cattle	Holstein	WssGWAS, RNA-seq	*PMAIP1*, *SBK1*, *TMEM33*, *GATB*, *CHORDC1*, *RTN4IP1*, and *BTBD7*	[193]
Adaptation	Cattle	Holstein	RNA-seq	*H1F1A*, *NR3C1*, and *ROCK1*	[194]
Adaptation	Cattle	Shanghai Holstein	selection signatures	*ITGA9*, *ACAT2*, and *PLAC8*	[197]
Adaptation	Poultry	Intercross line	GWAS	QTL on GGA chromosomes 1, 3, 6, 9, 10, 12–14, 17, 18, 21–28, and Z	[199]
Adaptation	Poultry	Guang Ming chickens	Transcriptome sequencing	*HSP90B1* and *HSPA5*	[200]
Adaptation	Poultry	Beijing You chickens’	Transcriptome sequencing	*CPT1A* and *ANGPTL4*	[200]
Adaptation	Cattle	Holstein	Whole metagenomic sequencing	Rhizobium and Pseudobutyrivibrio	[201]
Adaptation	Goat	Barbari goats	Gene expression	*HSP70*	[202]
Adaptation	Cattle	Holstein	SNP	*ATP1A1*	[90]
Adaptation	Cattle	Chinese Holstein cattle	SNP	*HSBP1*	[92]
Adaptation	Poultry	Huainan and Wenchang	SNP	*HSP90B1*	[91]
Adaptation	Buffalo	Nili ravi × Murrah	mRNA and miRNA expression	*IL18RAP*, *IL6R*, *CCR1*, *PPBP*, *IL1B*, and *IL1R1*	[103]
Adaptation	Sheep	Columbia × Rambouillet	GWAS	*FBX011*, *PHC3*, *TSHR*, and *STAT1*	[142]
Adaptation	Pig	Crossbred gilts	GWAS	*PAIP1*, *NNT*, *TEAD4*, *LIMS2*, *TTR*, *TEAD4*, *ERBB4*, *FKBP1B*, *NFATC2*, and *ATP9A*	[146]
Adaptation	Pig	Crossbred gilts	GWAS	*SSC12*, *SSC14*, *SSC16*, *SSC13*	[145]
Adaptation	Cattle	Gir × Holstein	GWAS	*LIF*, *OSM*, *TXNRD2*, and *DGCR8*	[148]
Adaptation	Poultry	Native chicken breed	Selection signature	*TSHR*	[149]
Adaptation	Buffalo	Azeri and Khusteni	Selection signatures	*FBXO9*, *NDFIP1*, *ACTR3*, *ARHGAP26*, *SERPINF2*, *BOLA-DRB3*, *BOLA-DQB*, *CLN8*, and *MYOM2*	[155]
Adaptation	Goat and Sheep	Barki	Selection signature	*FGF2*, *PLCB1*, and *GNAI3*	[154]
Adaptation	Pig	Xiang pigs	Selection signature	*PGDE1A*, *SDR9C7*, *UVSSA*, *NR2E1*, *SERPINB8*, *SLC2647*, *SERPINB10*, *MYO1A*, *EXPH5*, and *VEGFC*	[151]
Adaptation	Cattle	Abigar	Selection signature	*DNAJC18*, *HOXC13*, and *RXFP2*	[198]
Adaptation	Cattle	Tharparkar and Vrindavani	SNP	*ATP1A1*	[181]
Adaptation	Poultry	Broiler—Cobb500	Bisulfite sequencing	*TXNRD* and *Prdx1*	[195]

**Table 3:** Overview of identified biomarkers related to production and disease-resistant traits associated with heat stress.

Traits	Species	Breed	Methodology	Biomarkers	Reference
Disease resistance	Goat	West African dwarf, Red Sokoto and Sahel	SNP	*DRB*	[89]
Growth	Goat and sheep	Barki	Selection signature	*BMP2*, *BMP4*, *GJB2*, and *GJA3*	[154]
Growth and adaptation	Sheep	Barki and Aboudeleik	PCR–DNA sequencing	*CAST*, *LEP*, *MYLK4*, *MEF2B*, *STAT5A*, and *TRPV1*	[187]
Immune response	Poultry	Iranian indigenous chickens	Selection signature	*HSP70*, *HSPA9*, *HSPH1*, *HSP90AB1*, and *PLCB4*	[150]
Immune response and adaptation	Goat and Sheep	Barki	Selection signature	*IL2*, *IL7*, *IL21*, *IL1R1*, and *GRIA1*	[154]
Milk production	Cattle	Holstein	Whole transcriptomic sequencing	*TNF*, *IFNG*, and *IGF-1*	[100]
Milk production	Goat	Florida dairy goat	GWAS	*CSN3*, *ACACA*, and *ME1*	[143]
Milk production and adaptation	Cattle	Holstein cattle	GWAS	*TLR4*, *GRM8*, and *SMAD3*	[188]
Reproduction	Cattle	Holstein cattle	SNP	*AMH*, *IGFBP1*, *LRG5*, and *TLR4*	[190]
Reproduction	Cattle	Pasundhan	SNP	5′UTR region of HSP 70	[83]
Reproduction	Cattle		Transcriptomic analysis	*COPS5*, *HSPA8*, *PSMC6*, *POLR2L*, and *TPI1*	[191]
Reproduction	Pig	Duroc, landrace, and Yorkshire	SNP	The 5’ flanking region of the *HSP 70.2*	[82]

## Data Availability

No new data were created or analysed in this study. Data sharing is not applicable to this article.
